# Update on the current opinion, status and future development of digital pathology in Switzerland in light of COVID-19

**DOI:** 10.1136/jclinpath-2021-207768

**Published:** 2021-09-12

**Authors:** Viktor Hendrik Koelzer, Rainer Grobholz, Inti Zlobec, Andrew Janowczyk

**Affiliations:** 1 Medical Faculty, University of Zurich, Zurich, Switzerland; 2 Department of Pathology and Molecular Pathology, University Hospital Zurich, Zurich, Switzerland; 3 Institute of Pathology, Kantonsspital Aarau, Aarau, Switzerland; 4 Institute of Pathology, University of Bern, Bern, Switzerland; 5 Department of Oncology, Lausanne University Hospital, Lausanne, Switzerland; 6 Biomedical Engineering Department, Case Western Reserve University, Cleveland, Ohio, USA

**Keywords:** pathology, surgical, quality assurance, health care, telepathology

## Abstract

**Aims:**

The transition from analogue to digital pathology (DP) in Switzerland has coincided with the COVID-19 crisis. The Swiss Digital Pathology Consortium conducted a national survey to assess the experience of pathologists in dealing with the challenges of the pandemic and how this has influenced the outlook and adoption of DP.

**Methods:**

A survey containing 20 questions relating to DP, personal experiences and challenges during the pandemic was addressed to Swiss pathologists at different experience stages in private practice, community and university hospitals.

**Results:**

All 74 respondents were pathologists, with 81.1% reporting more than 5 years of diagnostic service experience. 32.5% reported having read 100 digital slides or more in a diagnostic context. 39.2% reported using whole slide imaging systems at their primary workplace. Key DP use cases before the COVID-19 lockdown were tumour boards (39.2%), education (60.8%) and research (44.6%), with DP used for primary diagnosis in 13.5%. During the COVID-19 crisis, the use of DP for primary diagnostics more than doubled (30% vs 13.5%), with internal consults as important drivers (22.5% vs 16.5%), while research use (25% vs 44.6%) and external consults (17.5% vs 41.9%) strongly decreased. Key challenges identified included a lack of established standard operating procedures and availability of specialised hardware and software.

**Conclusions:**

This survey indicates that the crisis acted as a catalyst in promoting DP adoption in centres where basic workflows were already established while posing major technical and organisational challenges in institutions that were at an early stage of DP implementation.

## Introduction

A previous study conducted in Switzerland by the Swiss Digital Pathology Consortium (SDiPath) suggested that the experiences and perspectives of Swiss pathologists concerning digitalisation are comparable with that of other reporting countries undergoing transitions to digital workflows.^1^ Now, 2 years later, and in the midst of a worldwide health crisis, a revised survey was conducted, in particular focusing on how Swiss clinical pathologists have been reacting to the crisis and how their views regarding digital and remote workflows have changed.

The motivation for conducting this survey is surrounded by the notion that digital pathology (DP) diagnostics are especially amenable to remote work. At a time when there are limited pathologists, and with the clinical need to ensure their continued ability to operate without being exposed to contagion risks, a confluence of factors potentially expediting the transition from traditional analogue to digital workflows are present. This survey was thus conducted to better understand if this transition has been hastened, as well as to understand any previously invisible limiting factors or changes in opinion as a result of novel experiences.

## Methods

A survey was developed by AJ, IZ, RG and VHK on behalf of the the SDiPath to include questions to characterise (1) the respondents according to their workplace, general diagnostic experience and experience with DP; (2) the extent of DP infrastructure at baseline, including the utilisation of whole slide imaging systems, image analysis, image management software (IMS) and laboratory information system (LIS) integration and specific use cases; (3) the utilisation of DP infrastructure during the COVID-19 crisis; and (4) specific challenges encountered at a personal, technical and organisational level. Question formats included yes/no answers, multiple choice and additional free-text fields for comments. A copy of the survey can be found in online supplemental material. The survey was implemented in Google Forms and all Swiss Society of Pathology (SSPath) and SDiPath members were notified by email; further, non-members were informed by SSPath and SDiPath members at their local institutions. Data were collected digitally over a 30-day time period starting from 2 June 2020 and continued until 2 July 2020; at that time the data collection phase was determined to be completed.

10.1136/jclinpath-2021-207768.supp1 Supplementary data

## Results

### Survey demographics

A total of 74 responses were received from the online survey, for a total response rate of 12.9% of all 572 staff or resident pathologists registered in Switzerland (18.6% of all 398 members of the SSPath). Of the respondents, 18.9% reported less than 5 years of diagnostic service experience, 23.0% 5–10 years, 24.3% 10–20 years and 33.8% more than 20 years of diagnostic experience. About one-third of the respondents (32.4%) have read more than 100 digital slides in a diagnostic context, with 8.1% reporting having read more than 1000 slides. Of the respondents, 39.2% indicated that a whole slide imaging system was in operation at their primary workplace, with 75.7% of these in place for less than 1 year in a diagnostic context. Of the respondents, 29.7% worked at a public or private community hospital, 35.1% at a university institute and 31.1% at a private laboratory.

### Uses of DP before and during the COVID-19 crisis

The key use cases for DP before the COVID-19 lockdown were reported for tumour boards (39.2%), education (60.8%) and research applications (44.6%), with DP used for primary diagnosis in 13.5%. Further, internal (16.2%) and external (41.9%) consults were common use cases for utilisation of DP. Of the respondents, 68.9% reported the availability of a slide scanner at their institution and the availability of viewing software in 66%. Image analysis for biomarker quantification (eg, breast cancer biomarkers) was used in 16.2% of cases. An IMS and LIS integration was available to 40.5%–50% of the respondents, respectively. In the crisis situation, DP was seen as a key use case for home office scenarios, with 56.8% of the respondents reporting that a home office scenario was considered. Of the respondents, 40.6% reported at least one pathologist at their institution working remotely, with 6.8% reporting more than five pathologists working from home office. In the remote sign-out situation, the use of DP strongly shifted from research, education and tumour board applications to primary diagnosis (30%, up from 13.5%) and internal consults (22.5%, up from 16.5%), while research use (25%, down from 44.6%) and external consults (17.5%, down from 41.9%) were greatly reduced in frequency. To enable remote diagnostics, 13.5% of the respondents reported systemic changes by their respective institutions in how the clinical patient information was accessed and/or integrated with the electronic medical record or the laboratory information system.

### Key challenges encountered

A key challenge for enabling remote sign-out was the need for the implementation of standard operating procedures (SOPs) to establish and validate the remote sign-out. Only 20.5% of the respondents using remote DP sign-out indicated that a standardised guideline or operating procedure was followed to self-validate diagnostic use. This lack of self-validation may be a consequence of tight schedules in the face of the pandemic but also the lack of established national guidelines for SOP validation of DP in Switzerland. In cases where no remote sign-out was possible, it was reported that a lack of an established digital workflow (51.4%), a lack of appropriate hardware (23%) and an insufficient network connection (16.2%) were key technical limitations preventing the utilisation of DP during the COVID-19 situation. Interestingly, soft factors such as lack of the usual work environment (21.6%), feeling uncomfortable with remote sign-out (13.5%) and concerns about data privacy and safety (10.8%) were other major reasons listed as keeping pathologists from implementing remote sign-out. To enable home office use, half of the respondents reported modest to strong support by the hospital information technology (IT) department, with over 70% reporting the need for material or set-up changes at the department, including a need for additional scanners (20.3%), high-resolution monitors (36.5%), viewing software (36.5%) and the set-up of virtual private network (VPN) or network connections (39.2%). Only 27% reported that no material or set-up changes would be required. Several solutions were implemented for the transmission of the generated reports into the LIS, including the remote connection to a dictation tool (28.4%), the generation of speech files sent to the dictation pool (17.6%) as well as self-writing of the reports for transfer to the clinicians (28.4%). Individual pathologists also used structured reporting (n=1) or communicated the results directly to the clinician (n=1), indicating that a unified solution was not available on short-term notice.

## Discussion

With the unexpected arrival of COVID-19 and its impact on routine pathology workflows, it is not surprising that hospitals worldwide attempted to better leverage DP in attempts at addressing their clinical needs. For example, a survey of tertiary UK hospitals reports^2^ indicated an increase in uptake of diagnostic DP during this period, supported by the implementation of remote access solutions and a fast roll-out of emergency guidance on how to risk-assess home reporting of digital slides by the Royal College of Pathologists.^3^ Italian regions with particularly severe COVID-19 case loads report the utilisation of DP solutions to support pathology work in the face of severe logistical constraints.^4^ It was recognised early that diagnostic delays could lead to a severe impact on public health and that continued operation of diagnostic services was critical to maintain timely diagnosis.^5^

Perhaps surprisingly, during times of crisis, it was indeed possible to compensate for the change in working environments needed using digital solutions. There was a large jump in the number of pathologists employing home office, and the number of primary diagnosis and consultations done via DP significantly rose. This seems to indicate that there is some latent infrastructure already in place at institutions which could support DP workflows. While perhaps no opportune time exists to search out and activate these resources, the observed reductions in case loads and the limited availability of alternative physical slide courier services appear to have strongly motivated their activation. In fact, a limited number of respondents are reporting systemic changes by their respective institutions (13.5%), and yet the overall number of pathologists employing remote sign-outs for diagnostics more than doubled in amount (30%, up from 13.5%). While it may be too early to fathom the long-term effects of the pandemic situation on pathology workflows, some have suggested that these changes may be permanent, introducing a new area of low-contact and high-interconnectivity pathology.^6^

Importantly, when thinking about ways to improve access and utilisation of DP in the future, availability of standards and regulations again appears towards the top of the list. In the crisis situation, national bodies reacted to the short-term need to use DP for routine diagnostics through the development of emergency guidelines^3^ or through the relaxation of government enforcement of the Clinical Laboratory Improvement Amendments to facilitate the utilisation of non-certified equipment to support parts of the workflow.^7^ In our case, although a majority of the pathologists reported not using SOPs, they still indicated their preference for at least obtaining them. This should not be surprising, especially in a time of crisis, that people forged ahead with their critical work in spite of a lack of definitive guidance, but indicates that as things return to normal and there is an opportunity to reflect on recent lessons, those SOPs should be codified and formally made available. To aid in this process, SDiPath has created a working group specifically for the creation of DP guidelines, including its usage in remote sign-out and consultation, which it intends to also publish and thus aid others in facilitating their own guideline creations.

While respondents generally indicated strong support from their institutions in getting set up to perform their work, technological hurdles did appear, which appeared to represent significant bottlenecks. Interestingly, many of these bottlenecks (eg, VPN connection, viewing software) can likely be easily ameliorated postcrisis with minimal effort.^8^ Given the experiences of their colleagues, and via word of mouth, it will be interesting to note in the future if an inflection point has been reached where the adoption of these technologies will be hastened to address potential future crises. Indeed, the lessons learnt from the COVID-19 pandemic underline how quickly the international community can collaborate to share best practices.^3^ It remains that additional training and education will likely be needed, not a finding specific to the crisis, but one that has been spoken about numerous times before. Some institutions used the crisis itself as an opportunity to provide such training, leading to the development of model teaching curricula that may lead to an increased exposure of trainees and residents to digital solutions going forward.^9 10^

A point brought out by our own survey and others has noted that DP remains dependent on the support of local IT, histolab, and scanner infrastructure and personnel.^11^ It is critical to not look past the fact that digital slides, although virtual, have paired physical samples which have been carefully prepared and manually introduced into the DP pipeline. Taken together, this survey and the opinions and results of other surveys again solidify the notion that DP is a multidisciplinary team endeavour and must be treated as such. In spite of the challenges identified and the bottlenecks encountered, importantly there appears to be an even more growing consensus that DP is a worthwhile investment and may sooner rather than later serve as an inevitable safeguard for future crises.

**Take home messages**

- The transition from analogue to digital pathology (DP) in Switzerland has coincided with the COVID-19 crisis; hence, the Swiss Digital Pathology Consortium conducted a national survey to assess the experience of pathologists in dealing with the challenges of the pandemic and how this has influenced the outlook and adoption of DP.
- We identify a confluence of factors expediting the transition from traditional analogue to digital workflows in ‘early adopter’ institutions, with a shift in the distribution of use cases from education and research to primary diagnostic use and consultation.
- At the same time, the crisis posed a major technical and organisational challenge in institutions that were at an early stage of digital pathology implementation, including a lack of established standard operating procedures, digital pathology workflows, and hardware and software equipment.
- This survey motivates the development and implementation of national guidelines led by the SDiPath to catalyse the experiences from the COVID-19 crisis into a safe usage of digital technologies.

10.1136/jclinpath-2021-207768.supp2 Abstract translation This web only file has been produced by the BMJ Publishing Group from an electronic file supplied by the author(s) and has not been edited for content.

10.1136/jclinpath-2021-207768.supp3 Abstract translation This web only file has been produced by the BMJ Publishing Group from an electronic file supplied by the author(s) and has not been edited for content.

10.1136/jclinpath-2021-207768.supp4 Abstract translation This web only file has been produced by the BMJ Publishing Group from an electronic file supplied by the author(s) and has not been edited for content.

## Data Availability

All data relevant to the study are included in the article or uploaded as supplementary information. The datasets generated during and/or analysed during the current study are available from the corresponding authors upon reasonable request.
