# Supplementary material for: Update on the current opinion, status and future development of digital pathology in Switzerland in light of COVID-19
Source: J Clin Pathol. 2021 Sep 12:jclinpath-2021-207768. doi: 10.1136/jclinpath-2021-207768 (PMC8440121; doi:10.1136/jclinpath-2021-207768)

The COVID-19 pandemic and its associated lockdown has invigorated debate regarding the digitalization of pathologist workplaces and home offices. The Swiss Digital Pathology Consortium (SDiPath, sdipath.ch) needs your help to assess the practice, safety, and validity of the use of a "home office" scenario.

If you plan to, or are currently using, remote sign-out, please participate in the following brief survey. Your experience will help craft federal responses for the promotion of the safe and effective use of this technology beyond the current pandemic. We appreciate your time and look forward to the productive engagement of all pathologists, laboratory and IT professional staff, for the collection of critical real-world data.

1. What is your current role?

- Pathologist
- IT
- Lab staff
- Administrator
- Procurement
- Other (please specify)

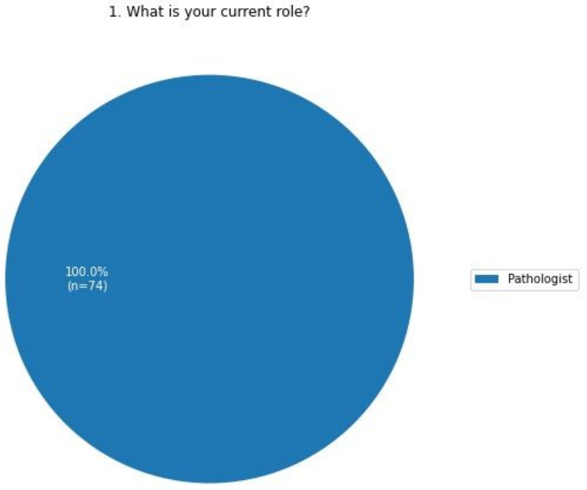

2. Where do you work?

- Hospital institute
- University institute
- Private institute
- Other

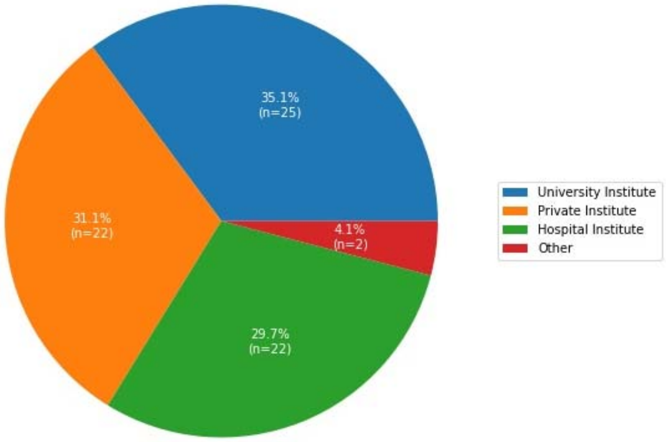

3. How many years have you been practicing (MDs) /involved with (lab staff) pathology?<br>• Up to 5 years<br>• 5-10 years<br>• 10-20 years<br>• More than 20 years	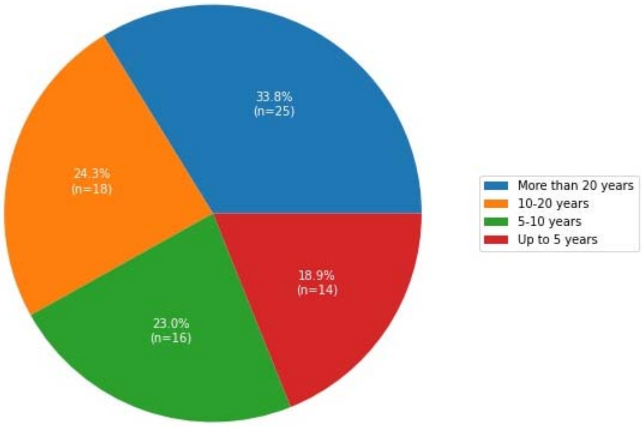

4. How many slides have you read digitally in your career so far in a clinical diagnostics context?<br>• 0<br>• 1-100<br>• 100-1000<br>• 1000-10000<br>• > 10000	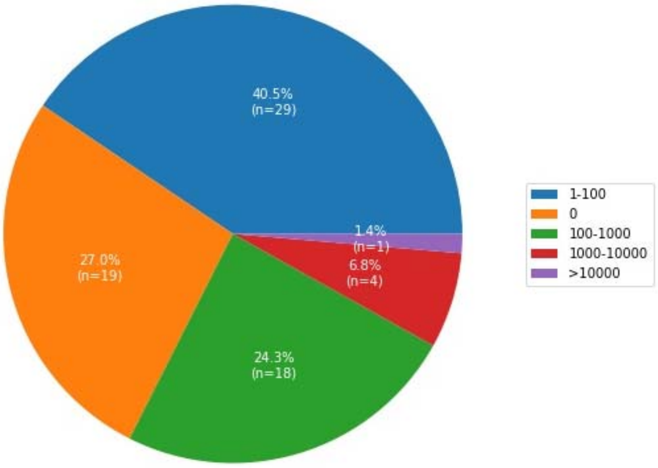

5. How many slides have you read digitally in your career so far in a research context?

- 0
- 1-100
- 100-1000
- 1000-10000
- 10000

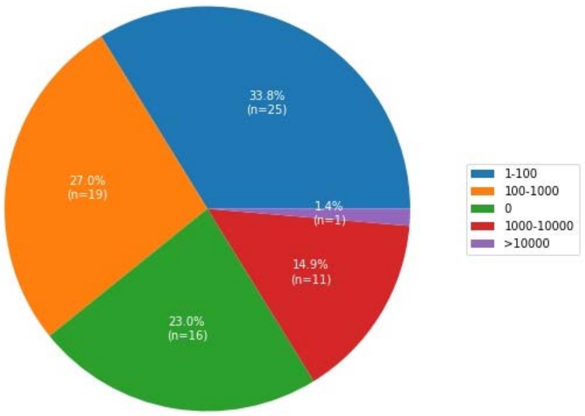

6. Are you currently using Whole Slide Imaging Systems (WSI systems, i.e. scanner, workstation, including display) and/or image analysis algorithms at your primary workplace, i.e. your hospital, lab, reference lab?

- Yes
- No

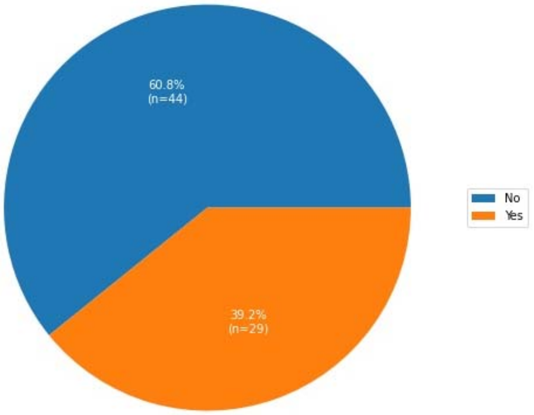

7. If yes, how many years have you been using WSI systems at your primary workplace?

- Less than 1 year
- Between 1 and 3 years
- More than 3 years

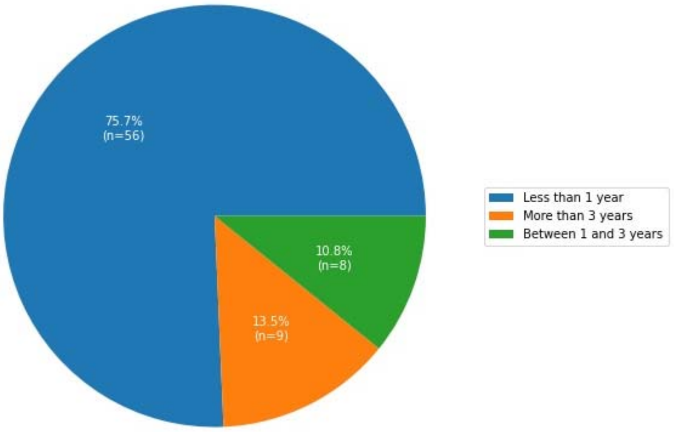

8. After a training phase, would your digital diagnostic workflow (open case/viewing slide/changing slide/ access to clinical information and previous results/ dictating and closing case) be...

- ...slower than analog reading of slides
- ...faster than analog reading of slides
- ...just as fast as reading analogue slides (no difference)
- ...don't have enough experience

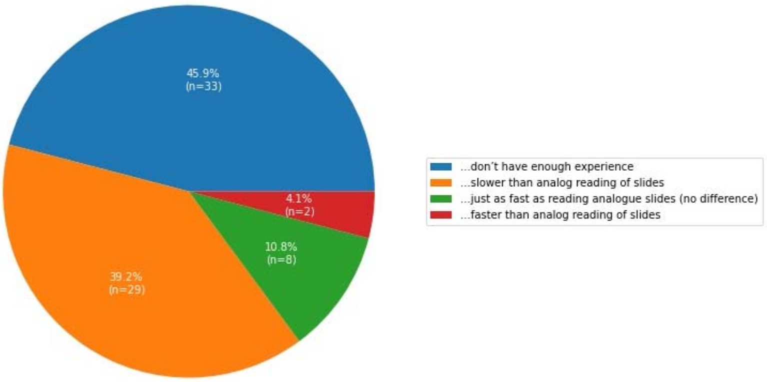

9. Before the COVID lockdown, what WSI use cases were you using at your institution in the last 6 months? (Please select all that apply)

a. Primary Diagnosis
b. External Consult, i.e. second opinion
c. Internal Consults, i.e. collaboration and or seeking colleague's expert opinion on a case before signing out
d. QC, e.g. check on batch controls, image quality, etc.
e. QA, e.g. correlation between frozen section and Primary Diagnosis (PDx), one pathologist confirming PDx of prior PDx, etc.
f. Education
g. Tumor Boards
h. Research
i. Image Analysis, such as breast markers, MMR
j. Other (please specify)

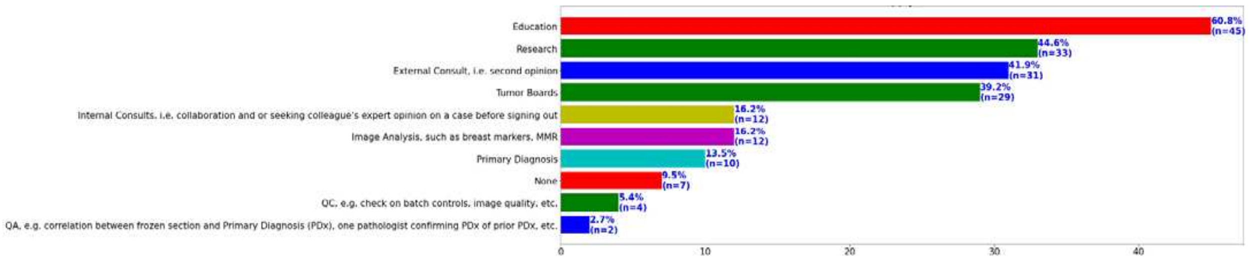

10. Before the COVID-19 lockdown, please identify which of the following products were used by your pathology system at your lab/hospital.

a. Scanner
b. Viewing Software
c. Image Management Software (IMS)
d. Lab Information System (LIS)

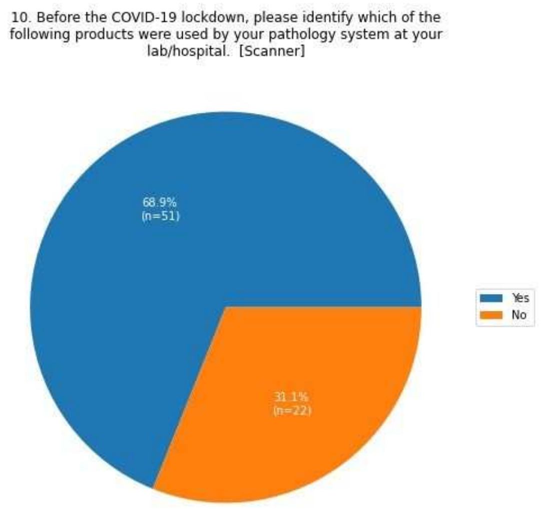

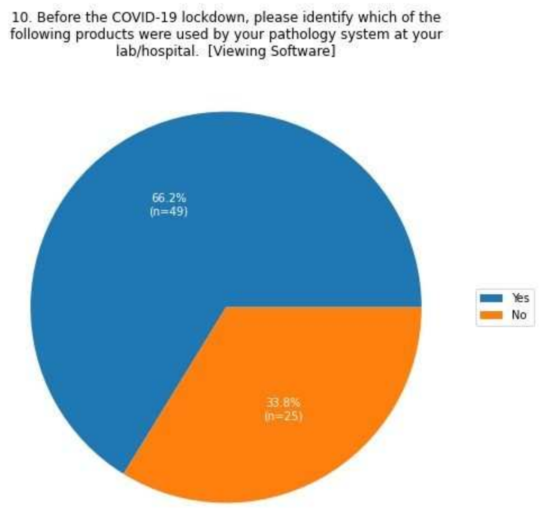

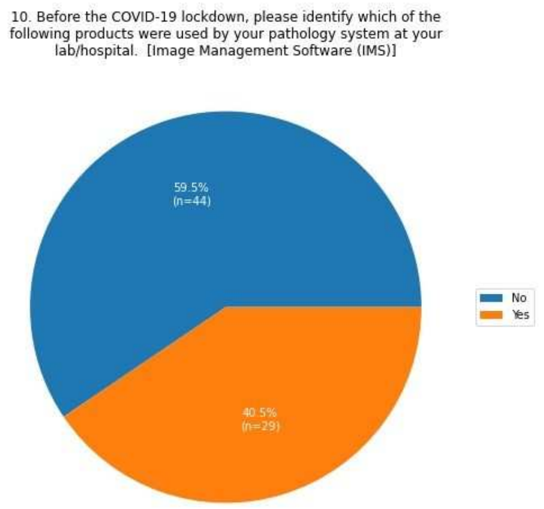

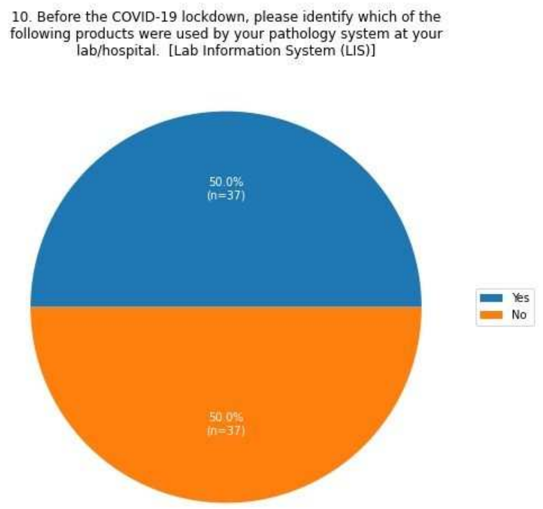

11. During COVID lockdown, did you consider a "home office" scenario for pathologists when the COVID lockdown started?

- Yes
- No

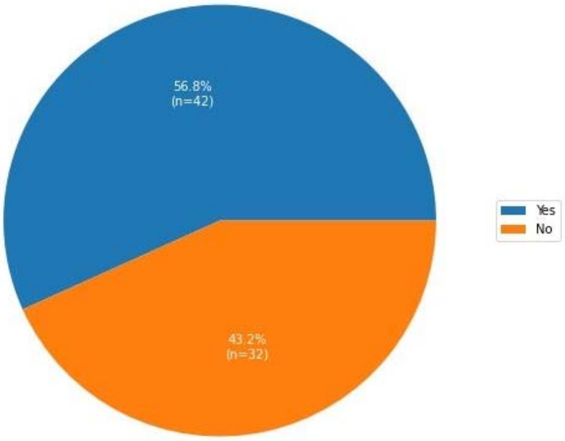

12. What level of support did you, or do you believe you would, have received from your IT department and/or system administrator for implementing a "home office"?

- very supportive
- modestly supportive
- limited support
- no support

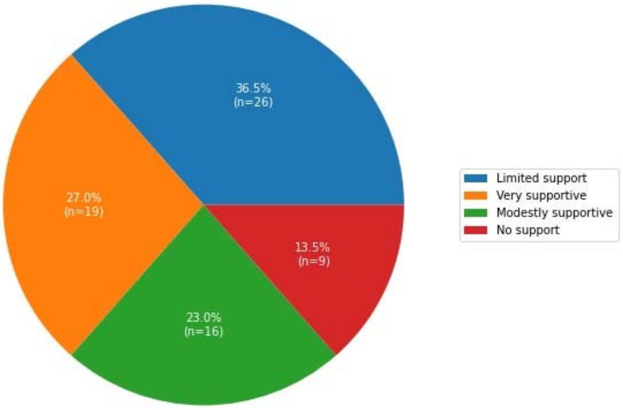

13. Did you require material or set-up changes to components of the Whole Slide Image System to enable “home office” during COVID lockdown?

Yes (Please select all that apply)

a. Scanner
b. Monitor
c. Viewing Software
d. VPN or Network Connection
e. Other (please specify) [text response]

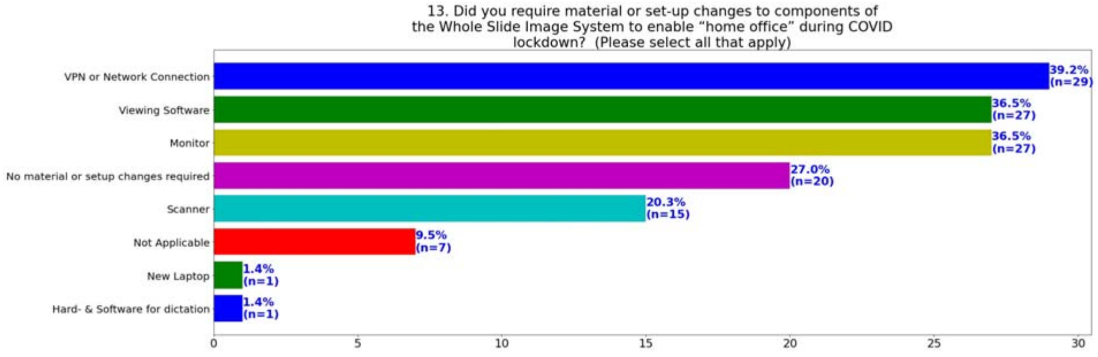

14. During the COVID lockdown, were you remotely signing out cases (Please select all that apply)?

a. Yes, for
   i. Primary Diagnosis
   ii. External Consult, i.e. second opinion
   iii. Internal Consults, i.e. collaboration and or seeking colleague’s expert opinion on a case before signing out
   iv. Research
   v. Image Analysis (such as breast markers, MMR…)
b. Not signing out cases

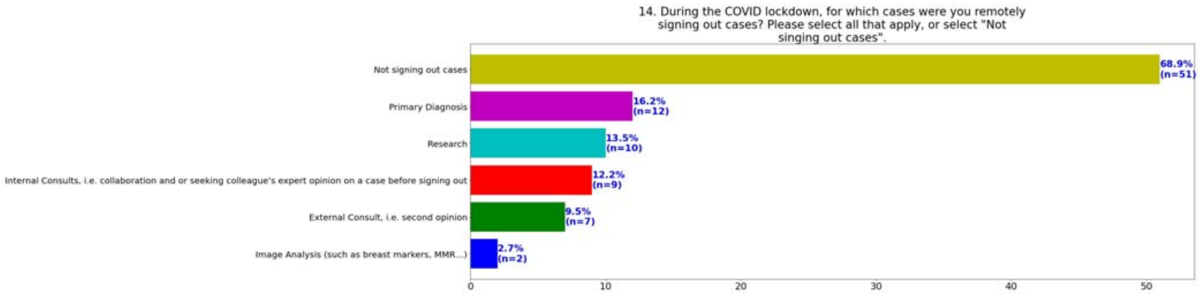

14 a. Subgroup analysis of use cases for pathologists remotely signing out cases during the COVID lockdown:

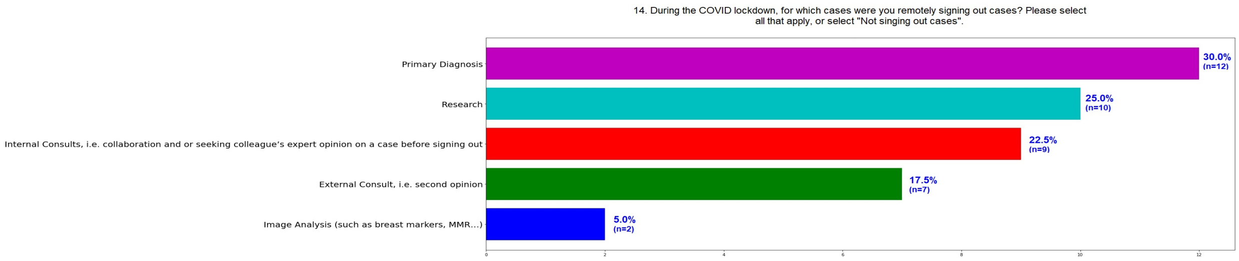

15. If no, what was preventing you from implementing remote sign out (multiple answers possible)?

a. Insufficient hardware at remote site
b. Insufficient network connection
c. Uncomfortable with the situation/fear of misdiagnosis
d. Workplace environment is missing (closeness to lab/colleagues/bibliography)
e. Concerns about data privacy / safety
f. No digital workflow set up
g. Other reason, please specify [text box]
h.

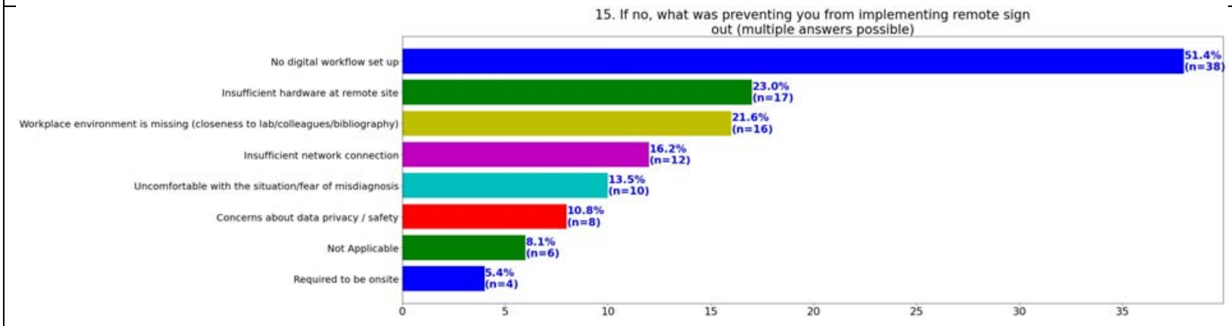

16. If diagnosing remotely ("home office"), how did (or would) you transmit your report into your Lab Information System (LIS) ?

a. Remote connection to dictation tool of the LIS
b. Generated speech file and sent to dictation pool
c. Wrote the report by myself
d. Other: [text]

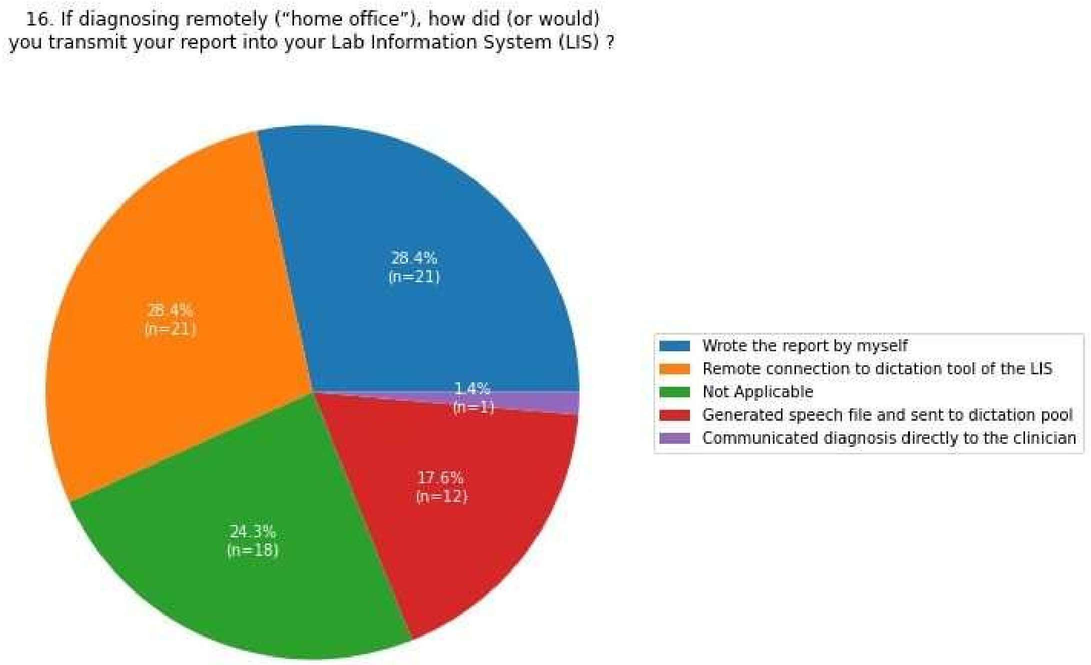

17. Did your institution change procedure(s) to access the clinical patient information during remote use such as how you access or integrate with the electronic medical record or the laboratory information system?

- Yes
- No

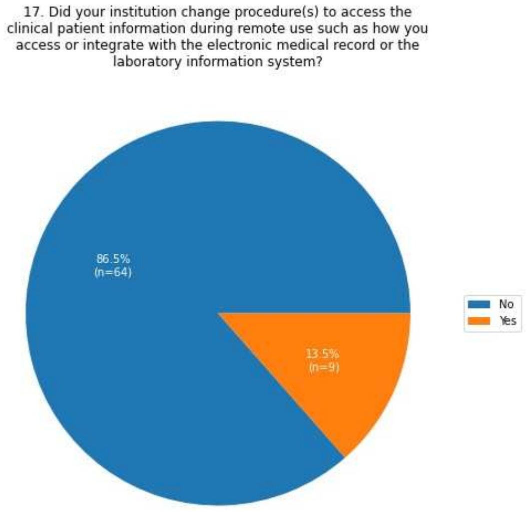

18. How many pathologists are/were signing out remotely at your institute?

- 0
- 1-4
- 5-9
- 10 or more
- Unknown

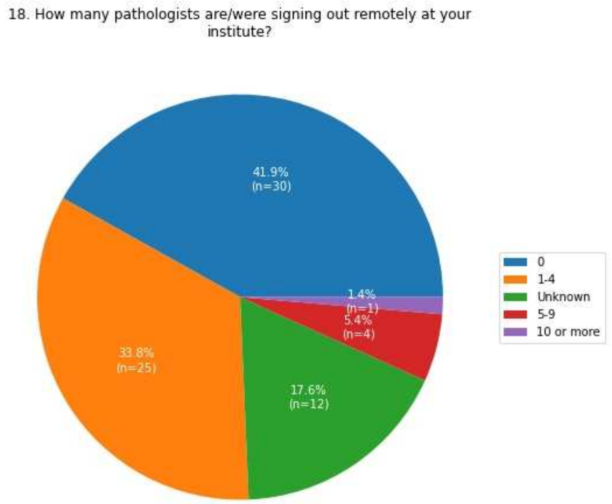

19. Have you used any guideline/internal SOP to self-validate the remote use sign-out?

- Yes
- No
- Unknown
- Do not use remote sign-out

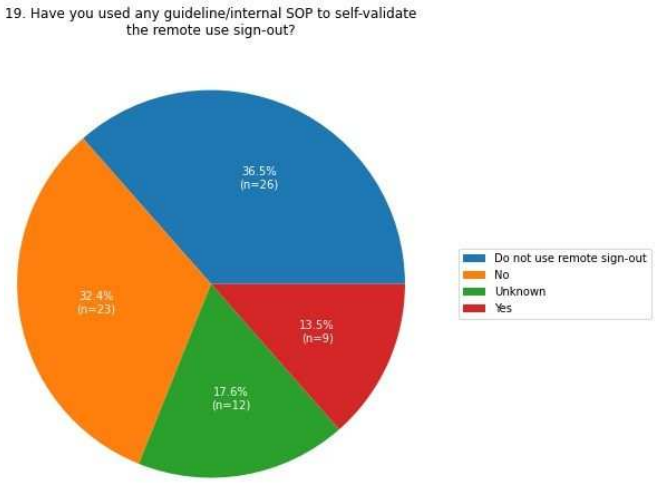

19 a. Subgroup analysis of guideline/internal SOP use within the subgroup of pathologists using remote signout?

- Yes
- No
- Unknown

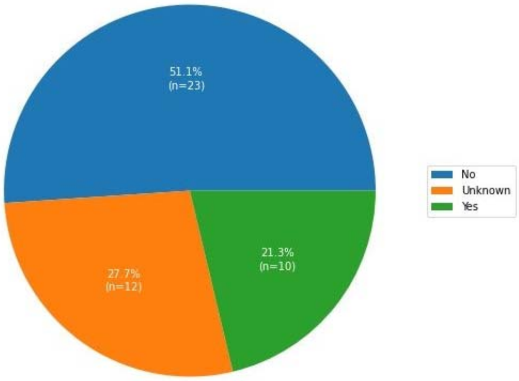

19b. If yes, is your institution willing to share its validation protocol and results?

- Yes
- No
- Unknown

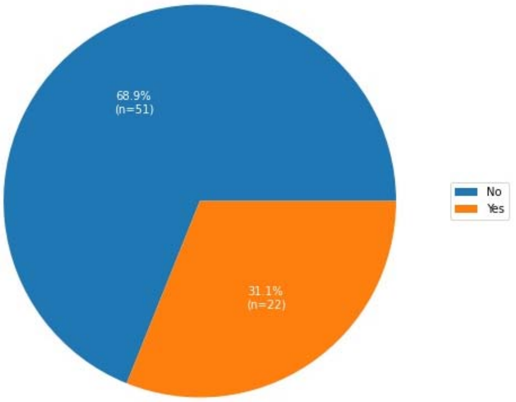

20. Has your institution included a quality assurance process such as retrospective review of a percentage of remotely signed out cases in the validation process?

- Yes
- No

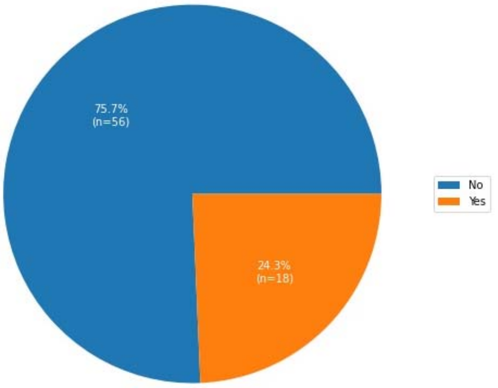

21. If regulations allowed, does your institution intend to continue to use or set up a remote sign-out (“home office”) following the COVID-19 pandemic?

- Yes
- In certain instances (e.g.
- shortage of staff)
- No

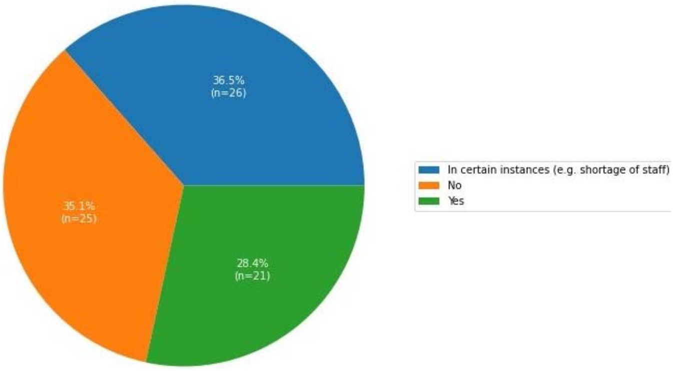

Supplement: Supplementary data [file jclinpath-2021-207768supp001.pdf]
