# Supplementary material for: Update on the current opinion, status and future development of digital pathology in Switzerland in light of COVID-19
Source: J Clin Pathol. 2021 Sep 12:jclinpath-2021-207768. doi: 10.1136/jclinpath-2021-207768 (PMC8440121; doi:10.1136/jclinpath-2021-207768)
Supplement: Abstract translation [file jclinpath-2021-207768supp002.pdf]

Supplemental material **10.1136/jclinpath-2021-207768**

This abstract has been translated and adapted from the original English-language content. Translated content is provided on an "as is" basis. Translation accuracy or reliability is not guaranteed or implied. BMJ is not responsible for any errors and omissions arising from translation to the fullest extent permitted by law, BMJ shall not incur any liability, including without limitation, liability for damages, arising from the translated text.

Objectifs: La transition de la pathologie analogique à la pathologie digitale (DP) en Suisse a coïncidé avec la crise du COVID. Le Consortium suisse de pathologie digitale (SDiPath) a mené une enquête nationale pour évaluer l'expérience des pathologistes face aux défis de la pandémie et comment cela a influencé les perspectives et l'adoption de la DP.

Méthodes: Une enquête contenant 20 questions relatives au DP, aux expériences personnelles et aux défis pendant la pandémie a été adressée à des pathologistes suisses à différents stades d'expérience en pratique privée, dans les hôpitaux communautaires et universitaires.

Résultats : Tous les n=74 répondants étaient des pathologistes, 81,1 % d'entre eux déclarant plus de 5 ans d'expérience en services de diagnostic. 32,5 % ont déclaré avoir lu 100 lames virtuelles ou plus dans un contexte de diagnostic. 39,2 % ont déclaré utiliser des systèmes d'évaluation d'images de lames entières sur leur lieu de travail principal. Les principaux cas d'utilisation du DP avant le confinement dû au COVID étaient les conférences clinico-pathologiques (39,2%), l'éducation (60,8%) et la recherche (44,6%) avec le DP utilisé pour le diagnostic primaire dans 13,5%. Pendant la crise COVID, l'utilisation de la DP pour les diagnostics primaires a plus que doublé (30 % contre 13,5 %), les consultations internes étant des moteurs importants (22,5% contre 16,5 %), tandis que la recherche et les consultations externes (17,5% contre 41,9%) ont fortement diminué. Les principaux défis identifiés comprenaient le manque de procédures opérationnelles standard établies et la disponibilité de matériel et de logiciels spécialisés.

Conclusion: Cette enquête indique que la crise a agi comme un catalyseur en favorisant l'adoption du DP dans les centres où les flux de travail de base étaient déjà établis tout en posant des défis techniques et organisationnels majeurs dans les institutions qui étaient à un stade précoce de la mise en œuvre du DP.
