# Supplementary material for: Update on the current opinion, status and future development of digital pathology in Switzerland in light of COVID-19
Source: J Clin Pathol. 2021 Sep 12:jclinpath-2021-207768. doi: 10.1136/jclinpath-2021-207768 (PMC8440121; doi:10.1136/jclinpath-2021-207768)
Supplement: Abstract translation [file jclinpath-2021-207768supp003.pdf]

Supplemental material **10.1136/jclinpath-2021-207768**

This abstract has been translated and adapted from the original English-language content. Translated content is provided on an "as is" basis. Translation accuracy or reliability is not guaranteed or implied. BMJ is not responsible for any errors and omissions arising from translation to the fullest extent permitted by law, BMJ shall not incur any liability, including without limitation, liability for damages, arising from the translated text.

Ziele: Der Übergang von der analogen zur digitalen Pathologie (DP) in der Schweiz fällt mit der COVID-Krise zusammen. Das *Swiss Digital Pathology Consortium* (SDiPath) führte eine nationale Umfrage durch, um den aktuellen Stand und das Potential für die zukünftige Entwicklung der digitalen Pathologie zu beurteilen.

Methode: Das *Swiss Digital Pathology Consortium* richtete eine Umfrage an Schweizer Pathologen unterschiedlicher Erfahrungsstufen in niedergelassenen, kommunalen und universitären Spitälern, um die verfügbaren Ressourcen, Verwendungszwecke und die Veränderungen der DP in der Krisensituation zu beurteilen.

Ergebnisse: Alle n=74 Befragten waren Pathologen, 81,1% berichteten von mehr als 5 Jahren Erfahrung in der Diagnostik. 32,5 % gaben an, 100 oder mehr digitale Objektträger in einem diagnostischen Kontext beurteilt zu haben. 39,2% gaben an, digitale Diagnostik an ihrem Hauptarbeitsplatz einzusetzen. Wichtige DP Anwendungsfälle vor der COVID-Krise waren Tumorboards (39,2%), Lehre (60,8%) und Forschung (44,6%), wobei DP in 13,5% für die Primärdiagnose verwendet wurde. Während der COVID-Krise hat sich der Einsatz digitaler Lösungen für die Primärdiagnostik mehr als verdoppelt (30 % vs. 13,5%). Interne Konsultationen stellten hierfür einen wichtigen Treiber dar (22,5 % vs. 16,5%), während die Verwendung der DP für Forschungszwecke (25 % vs. 44,6%) und externe Konsile (17,5% vs. 41,9%) stark rückläufig waren. Das Fehlen etablierter Standardarbeitsanweisungen und die Verfügbarkeit von spezialisierter Hard- und Software stellten in der Krise maßgebliche Limitationen für den flächendeckenden Einsatz digitaler diagnostischer Lösungen dar.

Schlussfolgerung: Diese Umfrage zeigt, dass die Krise als Katalysator der Einführung von digitalen Lösungen in Zentren gewirkt hat, in denen grundlegende Arbeitsabläufe bereits etabliert waren. In Einrichtungen, die sich in einem frühen Stadium der Digitalisierung befanden, stellte die Krisensituation hingegen eine große technische und organisatorische Herausforderung dar.
