# Supplementary material for: Update on the current opinion, status and future development of digital pathology in Switzerland in light of COVID-19
Source: J Clin Pathol. 2021 Sep 12:jclinpath-2021-207768. doi: 10.1136/jclinpath-2021-207768 (PMC8440121; doi:10.1136/jclinpath-2021-207768)
Supplement: Abstract translation [file jclinpath-2021-207768supp004.pdf]

Supplemental material **10.1136/jclinpath-2021-207768**

This abstract has been translated and adapted from the original English-language content. Translated content is provided on an "as is" basis. Translation accuracy or reliability is not guaranteed or implied. BMJ is not responsible for any errors and omissions arising from translation to the fullest extent permitted by law, BMJ shall not incur any liability, including without limitation, liability for damages, arising from the translated text.

Obiettivi: Il passaggio da patologia analogica a patologia digitale (PD) in Svizzera ha coinciso con la crisi del COVID. Il *Swiss Digital Pathology Consortium* (SDiPath) ha condotto un'indagine nazionale per valutare l'esperienza dei patologi nell'affrontare le sfide della pandemia e come ciò abbia influenzato le prospettive e l'adozione della DP.

Metodi: Un sondaggio contenente 20 domande relative alla PD, esperienze personali e sfide durante la pandemia è stato rivolto a patologi svizzeri in diverse fasi dell'esperienza in studi privati, ospedali comunitari e ospedali universitari.

Risultati: Tutti i n=74 intervistati erano patologi, ~~con~~ l'81,1% dei quali con più di 5 anni di esperienza nel servizio diagnostico. Il 32,5% ha riferito di aver letto 100 preparati digitali o più in un contesto diagnostico. Il 39,2% ha riferito di utilizzare sistemi di imaging di interi vetrini nel luogo di lavoro principale. I principali casi d'uso della PD prima del blocco COVID erano le consulenze sui tumori (39,2%), l'istruzione (60,8%) e la ricerca (44,6%) con la PD utilizzata per la diagnosi primaria nel 13,5%. Durante la crisi COVID, l'uso della PD per la diagnostica primaria è più che raddoppiato (30% contro 13,5%) con i consulti interni come driver importante (22,5% contro 16,5%), mentre l'uso per ricerca (25% contro 44,6%) e le consulenze esterne (17,5% vs 41,9%) sono fortemente diminuiti. Le sfide chiave identificate includevano la mancanza di procedure operative standard ben definite e la disponibilità di hardware e software specializzati.

Conclusione: Questo sondaggio indica che la crisi ha agito da catalizzatore nel promuovere l'adozione della PD nei centri in cui i flussi di lavoro di base erano già consolidati, ponendo al contempo grandi sfide tecniche e organizzative alle istituzioni che erano in una fase iniziale dell'implementazione della PD.
